# Bradykinin Protects Human Endothelial Progenitor Cells from High-Glucose-Induced Senescence through B2 Receptor-Mediated Activation of the Akt/eNOS Signalling Pathway

**DOI:** 10.1155/2021/6626627

**Published:** 2021-09-11

**Authors:** Yuehuan Wu, Cong Fu, Bing Li, Chang Liu, Zhi He, Xing-Er Li, Ailing Wang, Genshan Ma, Yuyu Yao

**Affiliations:** ^1^Department of Cardiology, Zhongda Hospital, School of Medicine, Southeast University, Nanjing, Jiangsu, China; ^2^Department of Clinical Laboratory, Zhongda Hospital, School of Medicine, Southeast University, Nanjing, Jiangsu, China; ^3^Department of Clinical Science and Research, Zhongda Hospital, School of Medicine, Southeast University, Nanjing, Jiangsu, China; ^4^Department of Obstetrics, Zhongda Hospital, School of Medicine, Southeast University, Nanjing, Jiangsu, China

## Abstract

**Background:**

Circulating endothelial progenitor cells (EPCs) play important roles in vascular repair. However, the mechanisms of high-glucose- (HG-) induced cord blood EPC senescence and the role of B2 receptor (B2R) remain unknown.

**Methods:**

Cord blood samples from 26 patients with gestational diabetes mellitus (GDM) and samples from 26 healthy controls were collected. B2R expression on circulating CD34^+^ cells of cord blood mononuclear cells (CBMCs) was detected using flow cytometry. The plasma concentrations of 8-isoprostaglandin F2*α* (8-iso-PGF2*α*) and nitric oxide (NO) were measured. EPCs were treated with HG (40 mM) alone or with bradykinin (BK) (1 nM). The B2R and eNOS small interfering RNAs (siRNAs) and the PI3K antagonist LY294002 were added to block B2R, eNOS, and PI3K separately. To determine the number of senescent cells, senescence-associated *β*-galactosidase (SA-*β*-gal) staining was performed. The level of mitochondrial reactive oxygen species (ROS) in EPCs was assessed by Mito-Sox staining. Cell viability was evaluated by Cell Counting Kit-8 (CCK-8) assays. Mitochondrial DNA (mtDNA) copy number and the relative length of telomeres were detected by real time-PCR. The distribution of human telomerase reverse transcriptase (hTERT) in the nucleus, cytosol, and mitochondria of EPCs was detected by immunofluorescence. The expression of B2R, p16, p21, p53, P-Ser^473^AKT, T-AKT, eNOS, and hTERT was demonstrated by Western blot.

**Results:**

B2R expression on circulating CD34^+^ cells of CBMCs was significantly reduced in patients with GDM compared to healthy controls. Furthermore, B2R expression on circulating CD34^+^ cells of CBMCs was inversely correlated with plasma 8-iso-PGF2*α* concentrations and positively correlated with plasma NO levels. BK treatment decreased EPC senescence and ROS generation. Furthermore, BK treatment of HG-exposed cells led to elevated P-Ser^473^AKT and eNOS protein expression compared with HG treatment alone. BK reduced hTERT translocation in HG-induced senescent EPCs. B2R siRNA, eNOS siRNA, and antagonist of the PI3K signalling pathway blocked the protective effects of BK.

**Conclusion:**

BK, acting through PI3K-AKT-eNOS signalling pathways, reduced hTERT translocation, increased the relative length of telomeres while reducing mtDNA copy number, and finally protected against EPC senescence induced by HG.

## 1. Introduction

Endothelial progenitor cells (EPCs) seem to play a key role in the repair of the endothelium and to improve neovascularization [1–3]. Premature senescence of EPCs related to gestational diabetes mellitus (GDM) can impair their function [4]. Ageing is a universal, progressive, and irreversible decline in function in tissue stem cells [5]. In addition, diabetes has been consistently associated with reduced circulating EPCs [6–9].

Nitric oxide (NO) has been described to reduce endothelial cell senescence, whereas NO blockade has an adverse effect, effects that are associated with telomeric DNA synthesis by telomerase [10, 11]. However, while high glucose (HG) can induce cell senescence via the NO-related pathway [12], the mechanism by which HG induces cord blood EPC senescence is unclear.

The tissue kallikrein-kinin system (KKS) triggers various beneficial effects, such as antihypertensive and antioxidant effects [13]. Kinins have a multitude of functions, including potent endothelium-mediated vasodilatory and antithrombotic properties. A previous study showed that B2 receptor (B2R) activation could be involved in the recruitment of circulating CD34^+^ cells with neovascularization potential [14]. Our previous study indicated that exogenous bradykinin (BK) significantly inhibited H_2_O_2_-induced EPC senescence via the B2R/AKT/RB and B2R/EGFR/RB signal pathways [15]. In addition, BK protects endothelial cells from H_2_O_2_-induced senescence through B2R-mediated NO release [16]. However, whether BK can protect EPCs from HG-induced senescence remains unclear.

This study was designed to investigate the detailed regulatory mechanism of the BK/B2R axis in preventing EPC senescence induced by HG.

## 2. Materials and Methods

### 2.1. Patient Groups

In total, we enrolled 26 GDM cases and 26 non-GDM controls in the analytical sample.

This study excluded pregnant females with certain risk factors, for example, women with in vitro fertilization or multiple pregnancies, inherited metabolic diseases, pregestational diabetes, or chronic hypertension. GDM is diagnosed according to WHO criteria. Women who had any of the following were regarded as having GDM: a 1 h 50 g glucose challenge test (GCT) ≥ 7.8 mmol/L, fasting plasma glucose of ≥7.0 mmol/L, 1 h oral glucose tolerance test (75 g OGTT) of ≥10.0 mmol/L, or 2 h OGTT ≥ 8.5 mmol/L. The work was approved by the Medical Ethics Committee of Zhongda Hospital affiliated with Southeast University (approval ID: 2018ZDSYLL069-Y01). All participants provided written informed consent.

### 2.2. Determination of B2R

The expression of B2R in the CD34^+^ circulating progenitor population of cord blood mononuclear cells (CBMCs) was determined by flow cytometry, as described in our previous studies [15].

### 2.3. Measurements of Plasma 8-Iso-PGF2*α* Levels and NO Levels

The 8-iso-PGF2*α* concentration was analysed using an 8-iso-PGF2*α* ELISA kit (CUSABIO, China). NO production in plasma was determined with an NO assay kit (Jiancheng Bioengineering Institute, Nanjing, China).

### 2.4. Assessment of HG-Induced Senescence and Mitochondrial ROS

EGM-2 MV medium is considered euglycaemic with a basal concentration of 5 mM D-glucose. EPCs were treated with either 40 mM D-glucose (Sigma, USA) alone or 1 nM BK (Sigma, USA) (given 30 minutes in advance) plus 40 mM D-glucose in 5% FBS-containing medium. Mannitol served as osmotic pressure control. The senescence of EPCs was determined by senescence-associated *β*-galactosidase (SA-*β*-gal) staining (BioVision). The SA-*β*-gal-positive cells were calculated and presented as percentage of the total cells. EPCs were incubated with Mito-Sox (Invitrogen). Fluorescence was evaluated in EPCs with a fluorescence microscope (Axio Observer A1, Germany). The acquired images were converted into binary images to quantify the average fluorescence intensity using ImageJ. EPC population viability was detected by the CCK-8 kit (Dojindo Laboratories). Total NO production in culture medium was detected using an NO assay kit (Jiancheng Bioengineering Institute, Nanjing, China).

### 2.5. Real-Time Quantitative Reverse Transcription PCR

The cells were lysed using TRIzol reagent, followed by synthesis of cDNA. p53, p21, and p16 mRNA levels were measured by real-time qRT-PCR, which was performed on a 7300 real-time PCR system (Applied Biosystems, Foster City, Calif., USA) using a SYBR green PCR kit (Thermo Fisher Scientific) according to the instruction manual. The primers are indicated in Table S1. p53, p21, and p16 mRNA expression was normalized to *β*-actin mRNA and calculated as 2^−∆∆Ct^.

### 2.6. Gene Silencing of B2R and eNOS Using Small Inhibitory RNA

The silencing protocol of B2R siRNA and eNOS siRNA was in accordance with the manufacturer's instructions as described previously [15]. The efficiency of specific B2R and eNOS siRNA inhibition was verified by Western blotting.

### 2.7. Determination of the Effect of B2R siRNA, eNOS siRNA, and PI3K Signalling Pathway Blockers on the BK Inhibition of HG-Induced Senescence and Mitochondrial ROS

The PI3K antagonist LY294002 (10 *μ*M) was added 5 minutes before BK treatment. The transfection of B2R siRNA and eNOS siRNA was performed as described above before BK treatment. EPCs were incubated with HG alone or with 1 nM BK as described above. Mannitol served as osmotic pressure control. Staining with SA-*β*-gal and Mito-Sox was performed as described above.

### 2.8. Western Blot Analysis

The protocol of protein extraction was in accordance with the manufacturer's instructions (Beyotime). Protein concentrations were measured by a BCA kit (Beyotime). Protein samples were loaded and separated by SDS-polyacrylamide gel electrophoresis before transfer to PVDF membranes. After blocking in TBST with 5% BSA, membranes were incubated overnight at 4°C with primary antibodies against AKT (1 : 2000, CST, USA), P-Ser^473^AKT (1 : 2000, CST, USA), eNOS (1 : 1000, CST, USA), p21 (1 : 1000, Abcam, UK), p16 (1 : 1000, Abcam, UK), B2R (1 : 1000, Abcam, UK), TERT (1 : 1000, Abcam, UK), p53 (1 : 1000, Santa Cruz, USA), and *β*-actin (1 : 1000, Abcam, UK). The PVDF membranes were subsequently incubated with florescent secondary antibody (1 : 10000, ZSGB, China). Finally, bands were developed with SuperSignal chemiluminescent substrate (Millipore, United States).

### 2.9. Measurement of Mitochondrial DNA (mtDNA) Content

Total DNA was extracted using the QIAamp DNA isolation kit (Qiagen, Hilden, Germany). mtDNA content was determined as previously described [17].

### 2.10. Real-Time PCR Detection of the Relative Length of Telomeres

The relative length of telomeres under different conditions was measured by quantitative PCR using the method described by Cawthon [18]. The ratio of the Ct value of the telomere gene to that of the 36B4 gene (a reference single copy gene) of each sample was designated the T/S ratio, which was used to reflect the relative length of telomeres.

### 2.11. Immunofluorescence Analysis of hTERT Translocation

EPCs were grown on coverslips within 12-well plates for 48 h prior to immunostaining. EPCs were treated as described above. After washing with PBS 3 times, EPCs were incubated with 50 nM/L MitoTracker Deep Red (Invitrogen) for 30 minutes at 37°C (5% CO_2_). Then, EPCs were fixed with 4% paraformaldehyde (30 min). Afterwards, the EPCs were permeabilized with 0.3% Triton X-100 (30 min). Next, the slides were incubated with anti-TERT primary antibody (Abcam) (1 : 50) overnight at 4°C. Then, the slides were incubated with Alexa Fluor-488 donkey anti-rabbit IgG (Invitrogen) (1 : 1000) for 1 h at room temperature. Finally, the slides were incubated with 4′-6-diamidino-2-phenylindole (10 min). Fluorescent images were recorded using a fluorescence microscope.

### 2.12. Statistics

Values were expressed as means ± SD. Data were analysed using one-way ANOVA followed by Bonferroni's multiple comparisons test. *P* values less than 0.05 were considered statistically significant.

## 3. Results

### 3.1. Flow Cytometric B2R Expression in CD34^+^ Cells of CBMCs from Newborns with or without GDM Mothers

**Figure 1:**
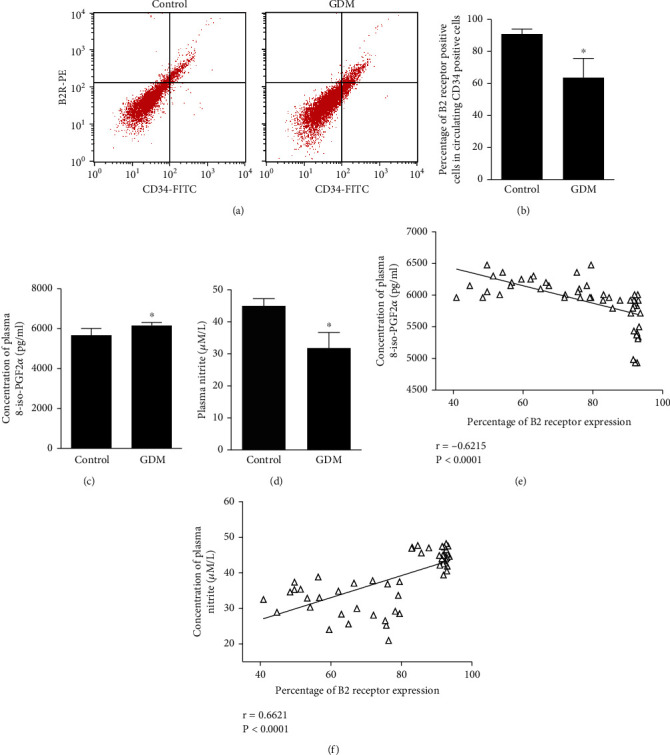
Expression of B2R in CD34^+^ cells of CBMCs from newborns with or without mothers with GDM. (a) Typical histograms of flow cytometric analysis of B2R expression in CD34^+^ cells of CBMCs from newborns with or without mothers with GDM. (b) Quantification of B2R expression by flow cytometry. (c) The 8-iso-PGF2*α* concentration was detected by a Direct 8-iso- PGF2*α* ELISA kit. (d) NO production was detected by NO assay kit. (e) Correlation between plasma 8-iso-PGF2*α* concentration and B2R expression. (f) Correlation between plasma NO concentration and B2R expression.

The characteristics of the study population are described in Table S2. In GDM newborns, B2R expression in CD34^+^ cells of CBMCs was lower than that in healthy controls (Figures 1(a) and 1(b)). As expected, increased 8-iso-PGF2*α* levels in the GDM newborn group were observed (Figure 1(c)). The plasma 8-iso-PGF2*α* concentration was significantly inversely correlated with B2R expression in CD34^+^ cells of CBMCs (*r* = −0.6215; *P* < 0.0001) (Figure 1(e)). An NO assay kit was used to assess NO production. Plasma level of NO production in GDM newborns was reduced compared to that in the non-GDM controls (Figure 1(d)). The plasma NO concentration correlated positively with B2R expression in CD34^+^ cells of CBMCs (*r* = 0.6621; *P* < 0.0001) (Figure 1(f)).

### 3.2. Induction of EPC Senescence

**Figure 2:**
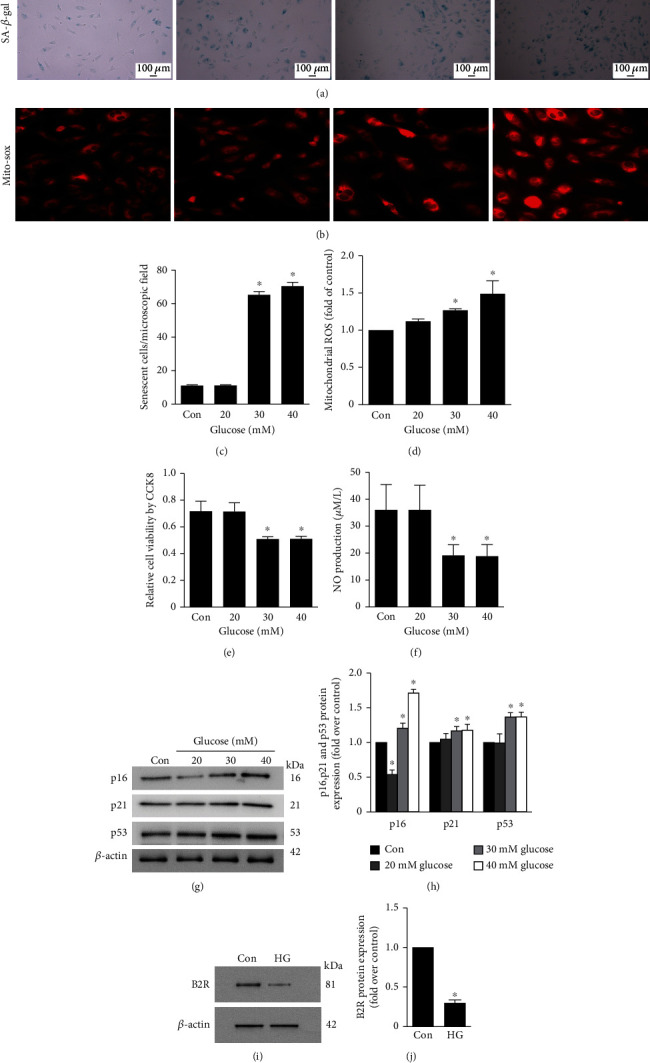
HG induced senescence in EPCs. (a) Representative photomicrographs of SA-*β*-gal staining in EPCs. (c) Number of senescent cells after treatment. (b, d) ROS generation was analysed by Mito-Sox. (e) Cell viability was evaluated by CCK-8 assay. (f) NO production was detected by NO assay kit. (g, h) Western blotting for p16, p21, and p53 in EPCs. (i, j) B2R protein levels 48 h after HG treatment. Bars represent the means ± SD (^∗^*P* < 0.05 versus control; *n* = 6).

EPCs have a typical cobblestone-like morphology. EPCs were stained for uptake of Dil-Ac-LDL or for lectin binding (Figure S1). We used 40 mM D-glucose, which did not induce apoptosis (data not shown), and incubated EPCs for 48 h. As demonstrated in Figures 2(a) and 2(c), incubation with HG significantly increased the percentage of SA-*β*-gal-positive senescent cells. HG induced an increase in mitochondrial ROS (Figures 2(b) and 2(d)). HG downregulated EPC proliferation (Figure 2(e)). Meantime, the NO secretion decreased (Figure 2(f)). To assess senescence, senescence-related protein expression was measured by Western blotting. p16, p21, and p53 levels were significantly increased 48 h after treatment with HG (Figures 2(g) and 2(h)). The level of B2R protein was lower in the HG group than in the control group (Figures 2(i) and 2(j)). The osmotic control with mannitol did not differ from the blank control (data not shown).

### 3.3. BK Protects against EPC Senescence

**Figure 3:**
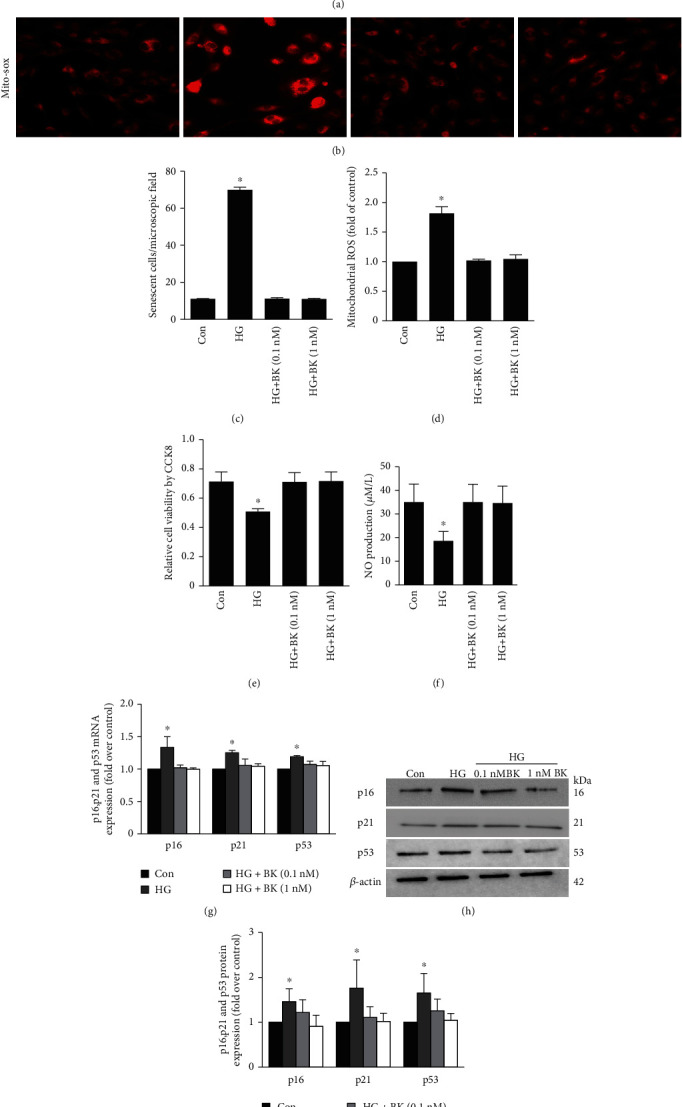
Effects of BK pretreatment on HG-induced EPC senescence. (a) Representative photomicrographs of SA-*β*-gal staining in EPCs. (c) Number of senescent cells after treatment. (b, d) ROS generation was analysed by Mito-Sox. (e) Cell viability was evaluated by CCK-8 assay. (f) NO production was detected by NO assay kit. (g) The mRNA expression of p16, p21, and p53 was detected by real-time PCR. (h, i) Western blotting for p16, p21, and p53 in EPCs. Bars represent the means ± SD (^∗^*P* < 0.05 versus control; *n* = 6).

EPCs were pretreated with 0.1 nM BK or 1 nM BK prior to HG treatment. As expected, BK administration reduced the EPC senescence rate and ROS generation (Figures 3(a)–3(d)). BK administration promoted EPC proliferation and NO secretion (Figures 3(e) and 3(f)). To assess senescence, p16, p21, and p53 mRNA and protein expression levels were measured. Levels of the p16, p21, and p53 mRNAs and proteins were significantly increased 48 h after treatment with HG. BK administration downregulated the expression of these mRNAs and proteins (Figures 3(g)–3(i)). BK had no effects on EPCs that were not treated with HG (data not shown). Induction of cell proliferation might confound the effects on p16, p21, and p53 mRNA and protein expression levels. Therefore, we tested the effect of 1 nM BK on the proliferation of EPCs and found no proliferative effect of BK after 48 h (data not shown).

### 3.4. BK Protects against EPC Senescence through B2R/PI3K/AKT/eNOS-Mediated NO Release

**Figure 4:**
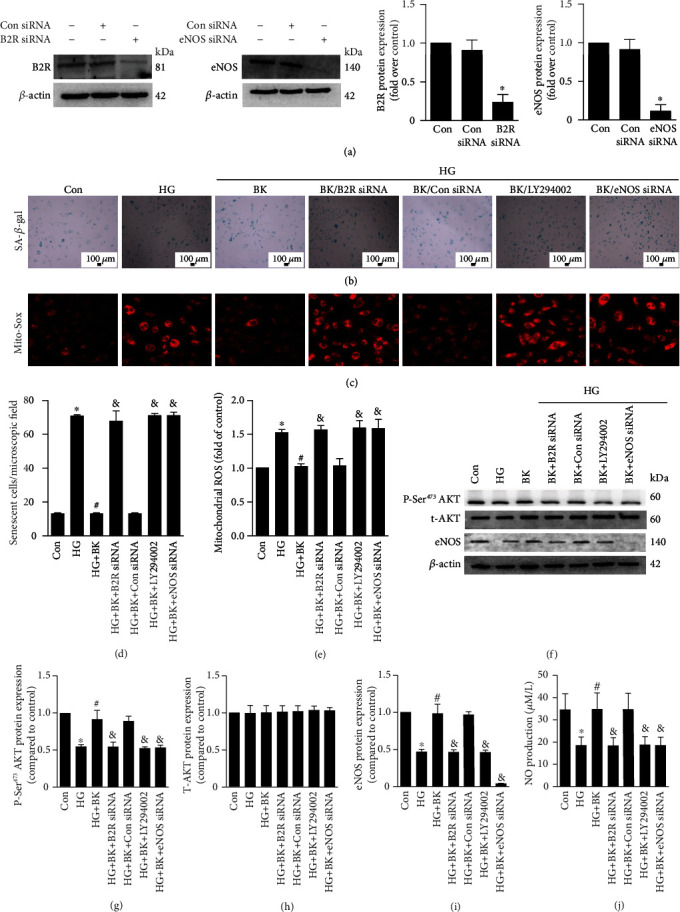
The role of B2R and NO in the protective effect of BK against senescence in EPCs. (a) Western blotting for B2R and eNOS in EPCs. (b) Representative photomicrographs of SA-*β*-gal staining in EPCs. (d) Number of senescent cells after treatment. (c, e) ROS generation was analysed by Mito-Sox. (f-i) Western blotting for P-Ser^473^AKT, T-AKT, and eNOS in EPCs. (j) NO production was detected by NO assay kit. Bars represent the means ± SD (^∗^*P* < 0.05 versus control; ^#^*P* < 0.05 versus HG; ^&^*P* < 0.05 versus HG + BK; *n* = 6).

To examine B2R silencing efficiency, samples from the control group and B2R siRNA group were subjected to Western blot analysis, and we observed decreased expression of B2R protein in the B2R siRNA group. Additionally, eNOS expression was downregulated in the eNOS siRNA group (Figure 4(a)). B2R siRNA, LY294002, and eNOS siRNA abrogated the protective effect of BK on EPC senescence and ROS generation (Figures 4(b)–4(e)). P-Ser^473^ AKT and eNOS levels were decreased 48 h after treatment with HG. BK fully prevented the change in P-Ser^473^ AKT and eNOS levels. HG decreased the NO release. BK prevented a decrease of NO release. As expected, B2R siRNA, LY294002, or eNOS siRNA treatment completely abrogated the beneficial effects of BK on P-Ser^473^ AKT and eNOS expression and NO release regulation induced by HG in EPCs. T-AKT expression was not altered (Figures 4(f)–4(j)). LY294002 had no effect in the absence of BK. Taken together, BK protects against EPC senescence through stimulation of B2R/PI3K/AKT/eNOS-mediated NO release.

### 3.5. BK Protects against EPC Senescence through Regulating hTERT Translocation

**Figure 5:**
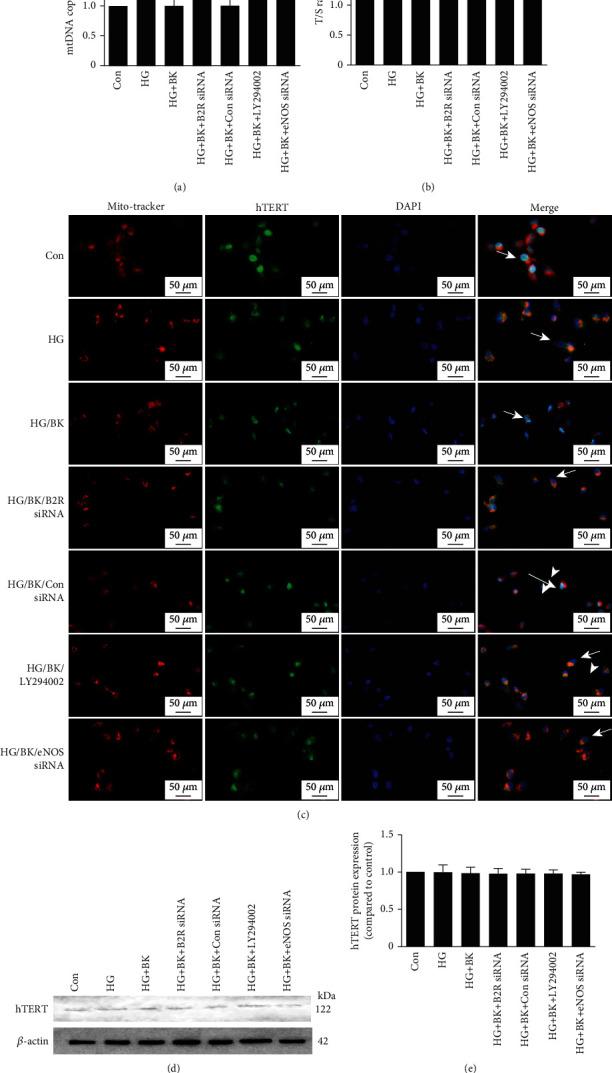
Effects of BK on mtDNA copy number, the relative length of telomeres, and hTERT translocation and hTERT expression in EPCs treated with HG. (a) The relative mtDNA content in EPCs was detected by real-time PCR. (b) The relative length of telomeres in EPCs was detected by real-time PCR. (c) Representative immunostaining is shown for the distribution of hTERT. Mitochondria, red fluorescence; hTERT, and green fluorescence. (d, e) Western blotting for hTERT in EPCs. Bars represent the means ± SD (^∗^*P* < 0.05 versus control; ^#^*P* < 0.05 versus HG; ^&^*P* < 0.05 versus HG + BK; *n* = 6).

Treatment with HG caused a significant increase in mtDNA copy number. In contrast, HG treatment decreased the relative length of telomeres in EPCs. Importantly, BK treatment prevented the increase in mtDNA copy number in EPCs treated with HG. Meanwhile, BK administration markedly inhibited the decrease in the relative length of telomeres induced by HG. As expected, B2R siRNA, LY294002, or eNOS siRNA treatment completely abrogated the beneficial effects of BK on mtDNA copy number and the relative length of telomeres induced by HG in EPCs (Figures 5(a) and 5(b)). In normal cells, hTERT is mainly located in the nucleus. In the immunofluorescence picture, we observed that the green fluorescence that represented hTERT was mainly located in the nucleus. After HG induction, the green fluorescence intensity was attenuated in the nucleus and enhanced significantly in the mitochondria. BK inhibited the decrease in nucleus and the increase in mitochondria of green fluorescence intensity, which indicated that BK inhibited hTERT translocation. However, incubation with B2R siRNA, LY294002, and eNOS siRNA inhibited the action of BK, indicating that the B2R-AKT-eNOS signalling pathway was involved in the effect of BK against HG-induced hTERT translocation (Figure 5(c)). In contrast, the hTERT protein level was not altered in whole cell lysates among all seven groups, demonstrating that the translocation of hTERT from the nucleus into the mitochondria preceded the downregulation of overall hTERT protein levels (Figures 5(d) and 5(e)).

## 4. Discussion

In this study, we showed that B2R expression in CD34^+^ cells of CBMCs was lower in women with GDM than in healthy women. This decrease was inversely correlated with high plasma levels of 8-iso-PGF2*α* and positively correlated with low plasma levels of NO. BK prevented EPC senescence via inhibition of hTERT translocation, which is characterized by increased relative length of telomeres and decreased mtDNA copy number in EPCs treated with HG. Importantly, BK regulated EPC hTERT translocation through the B2R-mediated Akt/eNOS signalling pathway. Therefore, we show here that BK protects against HG-induced EPC senescence through B2R-mediated NO release.

In this study, our results show that B2R expression is lower on circulating CD34^+^ cells of CBMCs from GDM women and is accompanied by higher degrees of 8-iso-PGF2*α* levels compared with healthy controls. Therefore, we hypothesize that B2R is the key element that regulates HG-induced CD34^+^ cell senescence. A previous study showed that the accumulation of ROS leads to accelerated senescence of cells, cell dysfunction, and arrest of the cell cycle at the G1 phase [19]. The mechanisms underlying HG-induced endothelial dysfunction have been extensively investigated. A previous study showed that carbonic anhydrase-I induced by HG hampers endothelial cell permeability and determines endothelial cell apoptosis in vitro [20]. Moreover, inflammatory factors participate in the progression of endothelial dysfunction caused by HG. Esposito et al. [21] reported the role of TNF alpha in endothelial dysfunction in the presence of HG. EPCs are bone marrow-derived and lineage-specific cells committed to becoming mature endothelial cells. The dysfunction of EPCs often leads to endothelial disorder. Patients with type 2 DM had lower EPC levels than healthy controls, similar to patients with other stress-related disorders, such as ischaemic heart disease. Indeed, during acute coronary syndrome and acute myocardial infarction, acute hyperglycaemia could cause a different response to primary angioplasty and DES stenting via downregulation of EPCs. This could condition a different extension of myocardial damage with negative effects on myocardial salvage and worse prognosis [22]. Adipose tissue is closely related to inflammation, and breast gland adipose tissue is estimated to account for different rates of major adverse cardiac events (MACEs) in premenopausal women [23, 24]. In this study, circulating CD34^+^ cells from 26 patients with GDM, along with samples from 26 BMI-matched healthy controls, were collected. We demonstrated that HG dose-dependently reduced the activity of EPCs and enhanced EPC senescence.

Previous studies showed that the PI3K/Akt/eNOS pathway was involved in the mechanism by which NO release reduced ROS generation [25, 26]. Senescent cells are irreversibly growth arrested and contain high expression of p21 and p16 cyclin-dependent kinase inhibitors [27]. Previous data indicated that p53 appears to play an important role in the regulation of p21 expression and senescence [28, 29]. In this study, BK exerted a protective effect against HG-induced ROS generation and thus reduced p53-mediated EPC senescence. The effect on EPC senescence may be attributed, at least in part, to inhibition of the PI3K-Akt-eNOS signalling pathway [30]. In this study, B2R siRNA, eNOS siRNA, and the PI3K inhibitor LY294002 completely abrogated the inhibitory effects of BK on EPC senescence induced by HG. These results suggest that BK prevents HG-induced EPC senescence by the Akt-eNOS-NO signalling pathway.

NO has the ability to protect against replicative senescence, effects that are associated with telomeric DNA repair by telomerase [10]. A variety of studies have demonstrated that the activity of TERT is essential to prevent cells from entering senescence by elongation of telomeres [31, 32]. Previous data demonstrate that hTERT was localized in the nucleus, cytosol and mitochondria of endothelial cells [33]. In this study, concomitantly with the significant increase in the formation of ROS in EPCs, nuclear hTERT protein was reduced, whereas mitochondrial hTERT protein increased. In contrast, hTERT protein was not altered in whole cell lysate. These results demonstrated that the translocation of hTERT from the nucleus into the mitochondria preceded the downregulation of overall hTERT expression. This is consistent with previous results reporting the mitochondrial translocation of TERT under increased ROS in vitro [34, 35]. In addition, the present results confirm that, in parallel to the increase in mitochondrial hTERT expression, mtDNA accumulates in a process that has been linked to mitochondrial malfunction [36] and to increased ROS [37–40]. Furthermore, BK stimulates NO release via the AKT/eNOS pathway and then further reduces ROS generation to maintain the length of telomeres under HG conditions. These results suggested that BK administration markedly inhibited EPC hTERT translocation and senescence induced by HG by reducing mitochondrial ROS dependent on B2R/AKT/eNOS-mediated NO release.

## 5. Limitations

There were some limitations, including that we used immunofluorescence rather than the telomeric repeat amplification protocol (TRAP) to evaluate hTERT protein levels, which may be less accurate. Further studies are needed to determine whether B2R overexpression can delay the progression of EPC senescence, and more detailed molecular mechanisms, such as a rather specific mechanism instead of an effect of ROS on the nuclear export of hTERT, remain to be further elucidated.

## 6. Conclusion

BK administration markedly inhibited EPC hTERT translocation and senescence induced by HG by reducing mitochondrial ROS dependent on B2R/AKT/eNOS-mediated NO release. Preventing the loss of B2R expression may represent a novel approach for the prevention and treatment of oxidative stress-related EPC senescence.

## Data Availability

The data used to support the findings of this study are available from the corresponding author upon request.

## Supplementary Materials

Supplementary 1 Figure S1: EPCs were stained for uptake of Dil-Ac-LDL or for lectin binding.

Supplementary 2 Table S1: the primers of EPCs used in RT-PCR. Table S2: clinical characteristic of enrolled pregnant.
