# Supplementary material for: Bradykinin Protects Human Endothelial Progenitor Cells from High-Glucose-Induced Senescence through B2 Receptor-Mediated Activation of the Akt/eNOS Signalling Pathway
Source: J Diabetes Res. 2021 Sep 11;2021:6626627. doi: 10.1155/2021/6626627 (PMC8452971; doi:10.1155/2021/6626627)
Supplement: Supplementary 1 — Figure S1: EPCs were stained for uptake of Dil-Ac-LDL or for lectin binding. [file 6626627.f1.pdf]

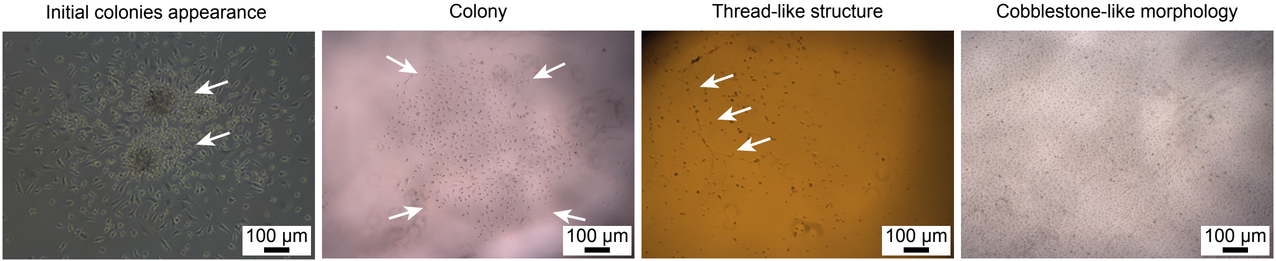
Initial colonies appearance
Colony
Thread-like structure
Cobblestone-like morphology
100 μm
100 μm
100 μm
100 μm

(a)

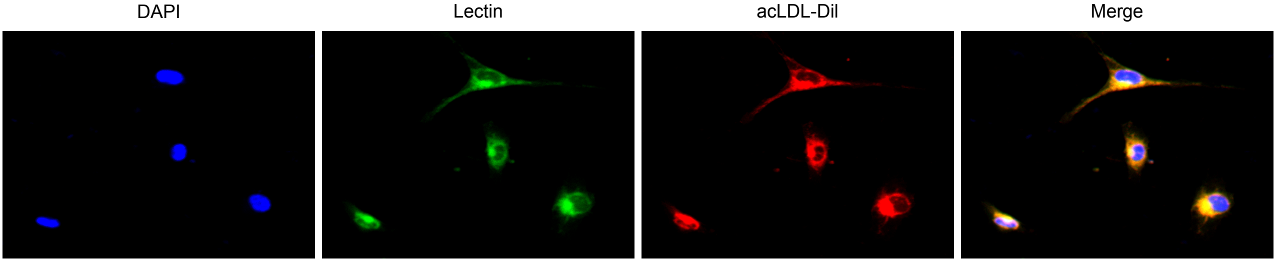
DAPI
Lectin
acLDL-Dil
Merge

(b)
