# Supplementary material for: Bradykinin Protects Human Endothelial Progenitor Cells from High-Glucose-Induced Senescence through B2 Receptor-Mediated Activation of the Akt/eNOS Signalling Pathway
Source: J Diabetes Res. 2021 Sep 11;2021:6626627. doi: 10.1155/2021/6626627 (PMC8452971; doi:10.1155/2021/6626627)
Supplement: Supplementary 2 — Table S1: the primers of EPCs used in RT-PCR. Table S2: clinical characteristic of enrolled pregnant. [file 6626627.f2.docx]

Table S1 The primers of EPCs used in RT-PCR

| Gene | accession number | Primer sequence (5’-3’) |
| --- | --- | --- |
| p16 | NM_000077 | F：ATCGCGATGTCGCACGGTA |
|  |  | R：CAATCGGGGATGTCTGAGGG |
| p21 | NM_001101 | F：GCGACTGTGATGCGCTAATG |
|  |  | R：GAAGGTAGAGCTTGGGCAGG |
| p53 | NM_000546 | F：GGTGCGTGTTTGTGCCTGTC |
|  |  | R：GGAGAGGAGCTGGTGTTGTTGG |
| β-actin | NM_001101 | F：AACCGCGAGAAGATGACCCAG |
|  |  | R：GGATAGCACAGCCTGGATAGCAA |

Table S2. Clinical characteristic of enrolled pregnant.

|  | **Control**  **n=26** | **GDM**  **n=26** | ***P value*** |
| --- | --- | --- | --- |
| Age (years) | 29.92±4.3 | 29.77±3.8 | NS |
| BMI | 27.48±2.2 | 27.46±3.0 | NS |
| Fasting plasma glucose (mmol/L) | 4.5±0.6 | 5.2±0.6 | 0.002 |
| HbA1c (%) | 5.7±0.2 | 7.2±1.4 | <0.001 |
| Albumin (g/L) | 34.5±2.7 | 36.4±4.7 | NS |
| Globulin (g/L) | 29.5±1.6 | 30.3±3.9 | NS |
| BUN (mmol/L) | 5.8±1.4 | 5.6±1.8 | NS |
| Scr (umol/L) | 57.3±9.6 | 56.6±11.4 | NS |
| Gestational age at delivery (wks) | 38.69±1.4 | 36.69±3.3 | NS |
| Birth weight (g) | 3527±269 | 3277±565 | NS |
| Microsomia (%) | 0 | 0 | / |
| Male baby (%) | 12 (46.2) | 12 (46.2) | / |
| Cesarean section (%) | 38.5 | 34.8 | NS |
